# Bevacizumab and Tocotrienol in Recurrent Platinum-Resistant Ovarian Cancer, and the Role of HOXA9 as a Prognostic Biomarker

**DOI:** 10.3390/diseases14030104

**Published:** 2026-03-12

**Authors:** Elisabeth Emanuel Graae, Louise Faaborg, Rikke Fredslund Andersen, Lars Ulrik Fokdal, Caroline Brenner Thomsen

**Affiliations:** 1 Department of Oncology, Lillebaelt University Hospital, Beriderbakken 4, 7100 Vejle, Denmark; 2 Department of Obstetrics and Gynecology, Aarhus University Hospital, Palle Juul-Jensens Boulevard 99, 8200 Aarhus, Denmark; loilrs@rm.dk; 3 Department of Biochemistry and Immunology, Lillebaelt University Hospital, Beriderbakken 4, 7100 Vejle, Denmark; rikke.fredslund.andersen@rsyd.dk; 4 Department of Oncology, Aarhus University Hospital, Palle Juul-Jensens Boulevard 99, 8200 Aarhus, Denmark; lars.fokdal@auh.rm.dk

**Keywords:** ovarian cancer, HOXA9, methylation, ctDNA, precision medicine, palliative treatment

## Abstract

Background/Objectives: Platinum resistant ovarian cancer represents a treatment challenge due to lack of efficient treatments and the absence of prognostic biomarkers. The circulating tumor DNA (ctDNA), methylated homebox A9 (meth-HOXA9), has been suggested as a biomarker for ovarian cancer, and might have a clinical impact in terms of predicting progression and supporting clinical decision making. Hence, this study investigated the prognostic value of meth-HOXA9 in platinum resistant recurrent ovarian cancer (PR-ROC) treated with bevacizumab and tocotrienol. Methods: Twenty patients with platin-resistant recurrent ovarian cancer were prospectively enrolled in this non-randomized phase II study. The treatment consisted of bevacizumab (Avastin) 10 mg/kg intravenously every three weeks and tocotrienol (Traptol) capsules 300 mg orally three times daily as a continuous treatment. The Level of meth-HOXA9 was measured at baseline and every three weeks. Results: The overall survival (OS) in the cohort was 7.5 months (95% CI 3.0–10.0), and the progression free survival was 4 months (95% CI 1.4–6.6). Comparing meth-HOXA9 ctDNA levels at baseline, there was no statistic significant difference in OS (*p* = 0.23). Conclusions: Treatment was well tolerated in this heavily pretreated cohort of PR-ROC patients with expected poor prognostic outcomes, with a few individuals showing extraordinary response in terms of progression free survival. The study was not powered to reproduce evidence of potential of meth-HOXA9 as a prognostic biomarker in PR-ROC.

## 1. Introduction

A recurrence of epithilial ovarian cancer (ROC) is most often diagnosed within two years after completion of platinum-based chemotherapy and characterized by short progression-free (PFS) and overall survival (OS). The response rate depends on the length of the treatment-free interval, as patients with early progression (≤6 months) after platinum-based chemotherapy have a poor prognosis with limited response rates ranging from 10 to 25% [1]. Platinum-resistant patients remain a therapeutic challenge and are often treated with several lines of palliative chemotherapy with potential severe toxicity. In this palliative setting, taking an unfortunate poor prognosis into consideration, the quality of life is crucial. So far, applicable biomarkers to select which patients may benefit from treatment have been an elusive goal, and there is an obvious need for new biomarkers for personalized treatment strategies. The monoclonal anti-angiogenic antibody bevacizumab is an integrated part of both first [2,3], second, and third line treatment in ovarian cancer [4,5]. Bevacizumab is well-tolerated, although there is a risk of side effects, including hypertension, proteinuria, gastrointestinal perforation, and an increased risk of thrombosis. Bevacizumab has been investigated in platinum-resistant epithelial ovarian cancer in the AURELIA trial. This study was the first open-label randomized phase III trial studying the effect of bevacizumab combined with cytotoxic chemotherapy in patients with platinum-resistant recurrent ovarian cancer (PR-ROC) [5]. Patients treated with bevacizumab supplementary to paclitaxel showed improved progression-free survival (PFS), with a median PFS of the chemotherapy group of 3.4 months, compared to 6.7 months in the group receiving bevacizumab (*p* = 0.001). Tocotrienols are parts of the natural E-vitamin. Several studies have reported a broad spectrum of biological activities of especially δ-tocotrienol, including effect of malignant cells. This effect is primarily proved in in vitro studies, but also in in vivo studies [6,7,8]. The δ-isoform, which appears to be particularly bioactive in oncologic settings, has been shown to downregulate vascular endothelial growth factor (VEGF) and to suppress endothelial cell proliferation, thereby impairing tube formation [9,10]. Reported toxicity is minimal to absent. Given that the anti-angiogenic properties of δ-tocotrienol likely contribute substantially to its anti-neoplastic activity, a complementary or additive interaction with bevacizumab can be hypothesized.

In ovarian cancer (OC), the use of circulating tumor DNA (ctDNA) as a biomarker has shown promising results, with emerging potential to support clinical decision making in more settings; namely, diagnostic, prognostic, monitoring treatment response, and in identification of therapeutic resistance [11]. The HOX genes and their role in cancers and potential as ctDNA biomarkers have been investigated in the last few decades. Multiple HOX genes are differently expressed in solid tumor tissue and vary from cancer type and location [12,13]. DNA methylation biomarkers, including methylation of the HOX genes, have been investigated in cohorts of ovarian cancer patients [14,15], and methylation of the HOXA9 gene has been found highly cancer-specific in ovarian cancer [16,17]. A previous phase II trial has investigated the effect of bevacizumab and tocotrienol with evaluation of HOXA9 methylated ctDNA (meth-ctDNA) in 23 patients with recurrent ovarian cancer [18]. The study found stable disease in 70% of the patients, and low toxicity. Moreover, a correlation between increasing meth-HOXA9 and poor prognostic outcome was seen. The prognostic potential of meth-HOXA9 has recently been investigated in several studies, all finding the presence and/or increase of meth-HOXA9 being negatively associated with PFS and OS [19,20,21,22]. The current open-labeled phase II trial study (Clinicaltrials.gov: NCT04175470, EudraCT no. 2019-000618-13) aims to access the survival in a heavily treated population, toxicity to bevacizumab and tocotrienol, and evaluate the effect of bevacizumab and tocotrienol in PR-ROC based on the level of HOXA9 meth-ctDNA.

## 2. Materials and Methods

### 2.1. Study Population

Patients with histologically verified PR-ROC treated with at least two different chemotherapeutic regiments were eligible for inclusion. Other inclusion criteria were: measurable disease by RECIST 1.1 criteria or evaluable by the GCIG CA125 criteria; age > or equal to 18; PS 0–2; adequate function of liver, bone marrow and kidney. Patients who met these predefined inclusion criteria were considered eligible. Those who, following a shared decision-making process, expressed a wish to receive further treatment were offered enrolment in the protocol. No additional selection criteria were applied. Patients were excluded if they had a history of other malignancies within three years prior to enrollment, with the exception of adequately treated basal cell carcinoma or squamous cell carcinoma of the skin. Additional exclusion criteria included: receipt of investigational treatment or participation in another clinical trial within 28 days before initiation of study therapy; underlying medical disease not adequately treated (diabetes, cardiac disease); clinically significant cardiovascular disease; bleeding tumor, surgery open biopsy, within 4 weeks prior to first dose of bevacizumab; uncontrolled hypertension; intestinal infiltration or infiltration in major blood vessels at the discretion of the treating physician; cerebral vascular attack within 6 months before start of treatment; allergies to active substance or any of the auxiliary agents. Further details and criteria are available in the protocol (Clinicaltrials.gov: NCT04175470, EudraCT no. 2019-000618-13). All patients were treated at the Department of Oncology, Vejle Hospital, during the period of March 2020 to October 2024. The trial was conducted in accordance with ICH-GCP guidelines and requirements. Written informed consent was obtained from all patients. All study data were recorded and administered using the Research Electronic Data Capture (REDCap) tool [23,24] hosted by Open Patient data Explorative Network (OPEN), Odense University Hospital, Region of Southern Denmark. Oversight and monitoring were performed by the regional Good Clinical Practice (GCP) unit. The investigation was conducted in accordance with the Reporting Recommendations for Tumor Marker Prognostic Studies (REMARK) criteria [25].

### 2.2. Treatment and Monitoring

A total of 20 patients received bevacizumab (Avastin) 10 mg/kg intravenously every three weeks and tocotrienol (Traptol) capsules 300 mg orally three times daily as a continuous treatment. Level of meth-HOXA9 was measured at baseline and three weeks after the first treatment cycle. Baseline HOXA9 meth-ctDNA levels and the levels at cycle two allowed for a division of the patients into two groups. The interpretation of HOXA9 meth-ctDNA levels are elaborated and applied in previously published papers [22,26]. A significant increase or decline in HOXA9 meth-ctDNA is defined beyond the 95% confidence interval, i.e., a significant change implies no overlap between the two 95% confidence intervals in comparison. The group with a significant increase in meth-HOXA9 level above the 95% confidence interval of baseline was allocated to Arm A and discontinued protocol treatment. Patients with stable or decreasing meth-HOXA9 continued protocol treatment in Arm B until progression of disease (PD), intolerable toxicity or patients’ wish for cease or condition not allowing further treatment. At inclusion a baseline CT scan was performed with identification of target lesions according to the Response Evaluation Criteria in Solid Tumours (RECIST) criteria. CT scans were performed following three treatment cycles. Blood samples including CA125 were collected for HOXA9 analysis at baseline and prior to each treatment cycle. Toxicity was graded and classified according to CTCAE version 4.0. PD was determined by an ovarian cancer expert (medical oncologist) by the RECIST and/or the GCIG CA125 criteria.

### 2.3. Analysis of HOXA9 Meth-ctDNA

Blood samples were collected at baseline and prior to every treatment cycle. The first two samples from each patient were analyzed prospectively to evaluate HOXA9 dynamics. Subsequent samples were stored at −80 °C. Blood was collected in 9 mL EDTA tubes and within four hours of sampling plasma was isolated by centrifugation (2000× *g*, 10 min). A second centrifugation was performed at 10,000× *g* for 10 min prior to analysis and exogenous control DNA (CPP1) was added as extraction control [27]. DNA was extracted from 4 mL of plasma using the QiaSymphony DSP Circulating DNA kit (Qiagen, Hilden, Germany) on a QiaSymphony SP instrument (Qiagen, Hilden, Germany). Bisulfite conversion was performed with the EZ DNA methylation lightning kit (Zymo Research, Irvine, CA, USA) according to the manufacturer’s instructions. Control samples included Universal human methylated control DNA (Zymo Research, Irvine, CA, USA), genomic DNA from whole blood and water. After conversion DNA was eluted in 15 µL and droplet digital PCR was performed on a BioRad QX200 system (BioRad, Hercules, CA, USA) for Albumin [28] and HOXA9 [29].

### 2.4. Statistical Analysis

Clinical variables were categorized and analyzed with descriptive statistics. Time to event analysis for progression and death was calculated from the start of treatment by means of actuarial Kaplan–Meier’s estimates. Patients were censored from time to event analysis in case of treatment-related morbidity that resulted in treatment discontinuation or at time of last follow-up. Comparison of Kaplan–Meier curves was done with a log rank test. All reported *p*-values were two-sided. The SPSS statistical software system v.20 (IBM SPSS Statistics for windows, Version 20.0, Armonk, NY, USA, IBM Corp.) was used for statistical analysis.

## 3. Results

### 3.1. Patient Characteristics

The study included 20 patients with advanced-stage ovarian cancer. In Table 1 the patient characteristics are presented. All patients had high-grade serous carcinoma. They were in performance status (PS) 0–2, with only one patient in PS 2. The patients were heavily pretreated, and the majority (75%) had received bevacizumab prior to inclusion.

### 3.2. Treatment Effect

**Figure 1:**
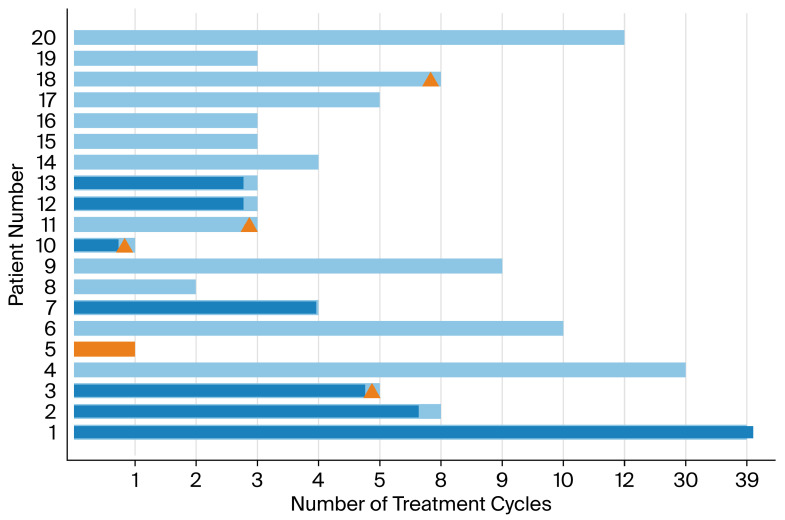
Toxicity, number of treatment cycles. Δ: Discontinuation due to toxicity or general condition. Dark blue: Significant decrease in meth-HOXA9 from baseline to after first treatment cycle. Orange: Significant increase in meth-HOXA9 from baseline to after first treatment cycle.

The median number of bevacizumab and tocotrienol cycles was four (range 1–39). Median PFS was 4 months (95% CI 1.4–6.6), and median OS was 7.5 months (95% CI 3.0–10.0). An estimated 25% of patients remained progression-free at 6 months. Two of these patients had remarkable response with 30 and 39 treatment cycles respectively before disease progression as seen in Figure 1.

### 3.3. Toxicity and Meth-HOXA9 Dynamics

In our study, two patients ceased treatment due to toxicity related to bevacizumab. These cases are further described in Table 2. Adverse events were CTCAE grade 2 nephrotoxicity, and CTCAE grade 3 epistaxis occurring after the first treatment cycle. No thromboembolism, gastrointestinal perforation or other serious adverse events were seen. Two patients ceased protocol treatment because of aggravation of their general condition. In these cases, contributing toxicity is possible. There were no treatment-related deaths.

**Figure 2:**
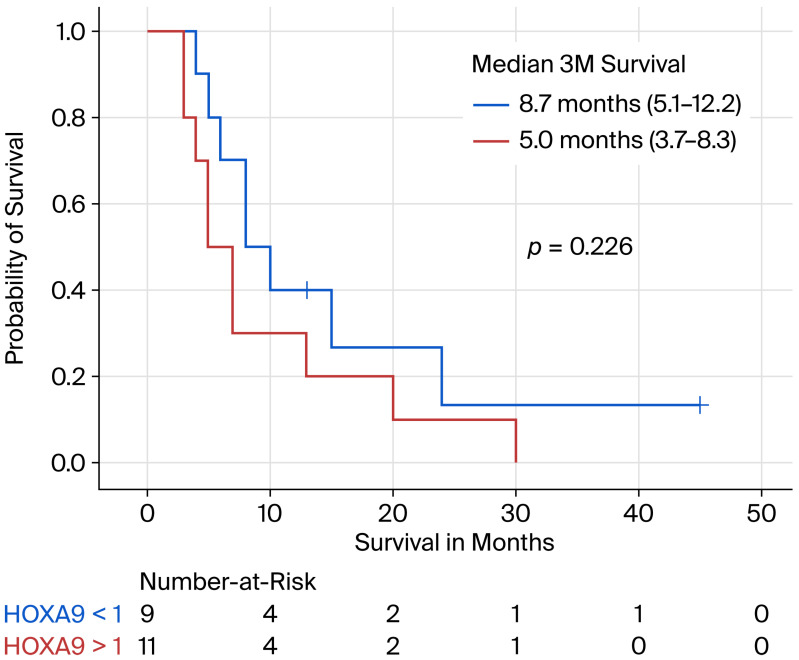
Overall survival and baseline meth-HOXA9 values <1 vs. >1.

In Figure 1, toxicity and meth-HOXA9 ctDNA dynamics are visualized, with dark blue color indicating significant decrease in meth-HOXA9 from baseline compared to the end of the first treatment cycle (*n* = 7) and orange color indicating significant increase (*n* = 1). Concerning meth-HOXA9, there was no tendency of increasing levels towards time of progression when comparing patients with a significant decrease in meth-HOXA9 levels to stable and rising levels, nor when evaluating meth-HOXA change from baseline to three weeks after the first treatment cycle. The patient (Arm A) having significantly increasing meth-HOXA9 had a poorer OS of 97 days. No difference was observed between the group of stable vs. decreasing meth-HOXA9 levels (*p* = 0.226). Baseline levels of meth-HOXA9 had no prognostic value in predicting OS, with a *p*-value of 0.23 comparing patients with high (>1) and low (<1) levels of meth-HOXA9, respectively (Figure 2). However, a non-significant statistical trend towards better OS in patients with low baseline meth-HOXA9 levels was observed, since patients with low baseline meth-HOXA9 levels had a median OS of 5 months vs. 8 months in patients with higher levels (>1).

## 4. Discussion

Patients with platinum-resistant ovarian cancer have a poor prognosis and there is an unmet need for effective treatments in this population. In the present phase II study, bevacizumab in combination with tocotrienol was investigated in heavily pretreated patients with PR-ROC. The general findings were low toxicity, median PFS of 4 months and OS of 7.5 months. The limitations of the current study imply a small study population and a long inclusion period of approximately 4.5 years. Survival data in our cohort showed results comparable to previous studies, taking the heavy disease burden into consideration. A single-agent phase II study of bevacizumab (gENENTECH avf 2949g) that included 44 patients with ovarian cancer with disease progression during or within 3 months after discontinuing topotecan or pegylated liposomal doxorubicin (PLD) showed a median PFS of 4.4 months (95% CI 3.1–5.5 months) and OS of 10.7 months [30]. Moreover, an estimated 28% of patients remained progression-free at 6 months, compared to 25% in our study. In a comparable phase II study [18] including highly pretreated chemotherapy refractory OC patients treated with bevacizumab and tocotrienol, median PFS and OS was 6.9 and 10.9 months, respectively, which is rather high compared to the current study. However, our cohort only included recurrent ovarian cancer (ROC). The AURELIA study found a median PFS of 3.4 months in patients treated with chemotherapy alone versus 6.7 months when treated with bevacizumab-containing therapy. Their median OS was 13.3 vs. 16.6 months, respectively [5]. In our study, OS was shorter, 7.5 months, but PFS was 4 months and thereby comparable to survival data from the group treated with chemotherapy alone in the AURELIA study, suggesting further evaluation of bevacizumab and tocotrienol as a treatment opportunity in ROC. In patients with ROC who have been treated with several treatment lines, further treatment options are limited, and therefore new strategies and more personalized treatments are warranted. Furthermore, heavily treated patients have accumulated toxicity which limits continuous treatment options. We found low toxicity in this study, which encourages further exploration. Future investigations should focus on enhancing clinical decision making by pointing out the patients who might benefit from non-cytotoxic treatment alternatives. Due to high recurrence rates and poor survival after relapse, there is an urgent need for early diagnosis of recurrence and prognostic biomarkers in OC. The widely used standard biomarker CA125 has limitations. Sensitivity of only 67.39% [31], latency in response not allowing reflection of treatment response after one or two treatment cycles, and the fact that around 20% of patients with ovarian cancer lack expression of the biomarker [31]. Circulating tumor DNA emerges as a promising method in this matter, bringing hope for more precise clinically valuable means of evaluating treatment response and early detection of recurrence [11,32,33,34]. The literature evaluating the clinical use of meth-HOXA9 in OC is limited, and the examined cohorts are generally small. A recent systematic review evaluating ctDNA as a biomarker for predicting PFS and OS in patients with epithelial OC, including 5 studies on the HOXA9 biomarker, concluded that high levels of ctDNA biomarkers were associated with worse prognostic outcomes [34]. The largest population size included in this systematic review on the HOXA9 biomarker is a study from our institution. A significant difference in OS in 126 ROC patients was found. The population, however, included platin-sensitive patients. A median OS of 7.5 and 24.7 months was measured, with detectable and undetectable meth-HOXA9, respectively [22]. Results from this prospective study of PR-ROC patients receiving bevacizumab and tocotrienol in a daily clinical setting indicate benefits comparable to the ones detected in earlier trials [5,18,30]. Despite the patients’ heavy cancer burden, the treatment was generally well tolerated.

No statistically significant differences in OS were observed according to baseline meth-HOXA9 levels or according to changes in meth-HOXA9 from baseline to completion of the first treatment cycle. However, given the limited sample size, the absence of statistical significance may reflect insufficient statistical power and an increased risk of a Type II error rather than a true lack of biological association.

As the study was exploratory and not powered to definitively assess the prognostic value of meth-HOXA9, these findings should be interpreted with caution. Nevertheless, the observed non-significant trend (Figure 2) may indicate potential clinical relevance and should be considered hypothesis-generating. Further investigation in larger, adequately powered cohorts of comparable patients is warranted to clarify the prognostic significance of meth-HOXA9 in this setting [15]. This could support clinical decision making in selecting the patients who are likely to benefit from therapy. Moreover, future research should focus on quality of life during therapy in advanced ovarian cancer.

## 5. Conclusions

Decisions about the appropriate treatment are complex. This study is considered a step towards personalized treatment, showing a low toxicity of bevacizumab and tocotrienol in a small cohort of patients with heavily pretreated PR-ROC. Although prognosis was expectedly poor, the benefits overall seemed to exceed the risks, and similarly to previous studies, we found an extraordinary response in specific individuals [20]. Prognostic value of the meth-HOXA9 marker could not be confirmed in our phase II trial.

## Figures and Tables

**Table 1:** Patient characteristics and treatment information.

Patient Characteristics	N = (%)
Age mean (range)	70 (55–86)
FIGO stage at diagnosis	
IIA	1 (5%)
IIIC	8 (40%)
IV	11 (55%)
Histology	
Serous high grade	20 (100%)
Number of prior regimens	
1–2	11 (55%)
3–4	5 (25%)
5–6	4 (20%)
Performance status	
0	13 (65%)
1	6 (30%)
2	1 (5%)
Prior treatment with bevacizumab	15 (75)
Number of treatment cycles on protocol treatment	
<4	8 (40%)
4–12	8 (40%)
>29	2 (10%)

**Table 2:** Treatment toxicity assessment by CTCAE version 4.0.

	Reason for Discontinuation Other Than Progression	Number of Treatment Cycles
Patient 3	Discontinuation for reasons of not feeling well reporting headache and subfebrilia. Additionally, elevation of liver parameters was observed.	5
Patient 10	Toxicity to bevacizumab: CTCAE grade 3 epistaxis. The patient was treated with one blood transfusion due to symptoms of anemia (hgb 5.7).	1
Patient 11	Discontinuation due to aggravation of general condition with fatigue and weight loss of 5 kg in a month. Stable disease on CT. CA125 10.000 (compared to 7600 at baseline)	3
Patient 18	Toxicity to bevacizumab: CTCAE grade 2 nephrotoxicity. Se-creatinin up to 217, and light hyperkalemia (4.7).	7

## Data Availability

The original data presented in the study are openly available in supplementary materials containing the data set used for synthesis. Further details and criteria are available in the protocol (Clinicaltrials.gov: NCT04175470, EudraCT no. 2019-000618-13).

## Supplementary Materials

The following supporting information can be downloaded at: https://www.mdpi.com/article/10.3390/diseases14030104/s1, File S1, The data set used for synthesis.

## Abbreviations

The following abbreviations are used in this manuscript:

PR-ROC	Platinum-resistant recurrent ovarian cancer
ctDNA	Circulating Tumor DNA
PFS	Progression-free survival
OS	Overall survival
PS	Performance status
